# Genome-wide CRISPR screen identified Rad18 as a determinant of doxorubicin sensitivity in osteosarcoma

**DOI:** 10.1186/s13046-022-02344-y

**Published:** 2022-04-23

**Authors:** Mingrui Du, Jintao Gu, Chenlin Liu, Nannan Liu, Zhe Yu, Chengpei Zhou, Wei Heng, Zhengcong Cao, Feilong Wei, Kailong Zhu, Yingwen Wang, Wei Zhang, Xiaochang Xue, Yong Zhang, Jixian Qian

**Affiliations:** 1 Department of Orthopedics, The Second Affiliated Hospital, The Fourth Military Medical University, Xi’an, China; 2 State Key Laboratory of Cancer Biology, Biotechnology Center, School of Pharmacy, The Fourth Military Medical University, Xi’an, China; 3 Experimental Teaching Center of Basic Medicine, The Fourth Military Medical University, Xi’an, China; 4 Preclinical School of Medicine, The Fourth Military Medical University, Xi’an, China; 5 National Engineering Laboratory for Resource Development of Endangered Crude Drugs in Northwest China, College of Life Sciences, Shaanxi Normal University, Xi’an, 710062 China; 6 The Key Laboratory of Medicinal Resources and Natural Pharmaceutical Chemistry (Shaanxi Normal University), The Ministry of Education, College of Life Sciences, Xi’an, China

**Keywords:** Genome-wide CRISPR screen, Rad18, Osteosarcoma, RGD-exosome

## Abstract

**Background:**

Osteosarcoma (OS) is a malignant bone tumor mostly occurring in children and adolescents, while chemotherapy resistance often develops and the mechanisms involved remain challenging to be fully investigated.

**Methods:**

Genome-wide CRISPR screening combined with transcriptomic sequencing were used to identify the critical genes of doxorubicin resistance. Analysis of clinical samples and datasets, and in vitro and in vivo experiments (including CCK-8, apoptosis, western blot, qRT-PCR and mouse models) were applied to confirm the function of these genes. The bioinformatics and IP-MS assays were utilized to further verify the downstream pathway. RGD peptide-directed and exosome-delivered siRNA were developed for the novel therapy strategy.

**Results:**

We identified that E3 ubiquitin-protein ligase Rad18 (Rad18) contributed to doxorubicin-resistance in OS. Further exploration revealed that Rad18 interact with meiotic recombination 11 (MRE11) to promote the formation of the MRE11-RAD50-NBS1 (MRN) complex, facilitating the activation of the homologous recombination (HR) pathway, which ultimately mediated DNA damage tolerance and leaded to a poor prognosis and chemotherapy response in patients with OS. Rad18-knockout effectively restored the chemotherapy response in vitro and in vivo. Also, RGD-exosome loading chemically modified siRad18 combined with doxorubicin, where exosome and chemical modification guaranteed the stability of siRad18 and the RGD peptide provided prominent targetability, had significantly improved antitumor activity of doxorubicin.

**Conclusions:**

Collectively, our study identifies Rad18 as a driver of OS doxorubicin resistance that promotes the HR pathway and indicates that targeting Rad18 is an effective approach to overcome chemotherapy resistance in OS.

**Supplementary Information:**

The online version contains supplementary material available at 10.1186/s13046-022-02344-y.

## Background

Osteosarcoma (OS) is a malignant bone tumor occurring in the metaphysis of the long diaphysis and is common in children and adolescents. The treatment is comprehensive limb salvage therapy with the combination of chemotherapy and surgery [1]. Osteosarcoma has intrinsic resistance to chemotherapy, and the effective rate of the first-line chemotherapy drugs cisplatin and doxorubicin is less than 30%. In addition, OS patients who receive chemotherapy for months quickly acquire resistance, with a rescue rate of only 20% in relapsed patients [2]. Although chemotherapy improves the prognosis of patients with OS, chemotherapy resistance is still one of the main reasons for treatment failure. Doxorubicin is a first-line chemotherapy drug for OS and a key drug for neoadjuvant and adjuvant chemotherapy. Therefore, reducing or even reversing doxorubicin resistance has become the key to improving chemotherapy outcomes in OS.

The occurrence of drug resistance in OS is regulated by multiple factors and multiple genes at multiple levels; the related changes include changes in drug transport-related pathways, changes in apoptotic pathways, changes in the cell microenvironment, enhanced DNA repair capacity and oxidoreductase activity. ABC transporter superfamily members, multidrug resistance-associated proteins, cytochrome p450 metabolic pathways and so on have been confirmed to be related to doxorubicin resistance in OS, but it is worth mentioning that no new targeted drugs have been developed to reverse drug resistance [3, 4]. Therefore, it is particularly important to find new drug resistance targets for OS and reverse drug resistance.

Recent innovations in genome editing technology, especially the emergence of the CRISPR/Cas9 system, have greatly promoted research on the functional genome of mammalian cells [5]. High-throughput second-generation genomic screening methods have been widely used to investigate the molecular mechanisms associated with specific cell phenotypes, including cancer chemotherapy resistance [6, 7]. The CRISPR/Cas9 system is increasingly applied to the study of gene function deficiency in various biological systems due to its easy programming and high gene editing efficiency [8, 9]. Compared with previous functional deletion screening systems, the CRISPR/Cas9 screening system has higher sensitivity, higher data reproducibility and minimal off-target effects. In recent years, CRISPR/Cas9 library screening has been used in various models to identify genes that are critical for cancer cell survival, proliferation, migration, and drug resistance [10–13].

In this study, we systematically evaluated the mechanism driving doxorubicin resistance in OS by genome-wide CRISPR/Cas9 knockout screening in OS cells treated with doxorubicin and DMSO (control). Subsequently, through comparative analysis of transcriptomic sequencing results of drug-resistant strains, we found that Rad18 E3 ubiquitin protein ligase (Rad18) may play a key role in the process of doxorubicin resistance in OS. Studies have found that Rad18 is involved in multiple genomic maintenance pathways. In addition to regulating the translesion synthesis pathway (TLS) by promoting PCNA monoubiquitination at k164 site [14, 15], Rad18 is also involved in the activation of Fanconi Anemia pathway [16] and interstrand cross-link repair pathway [17]. Our studies found that Rad18-knockout sensitized OS cells to doxorubicin in vivo and in vitro. Rad18 can interact with meiotic recombination 11 (MRE11), promote the formation and function of the MRE11-RAD50-NBS1 (MRN) complexes, further promote the homologous recombination (HR) pathway of DNA double-strand breaks (DSBs), and mediate doxorubicin resistance in OS. In the orthotopic transplantation OS nude mouse model, combined with doxorubicin chemotherapy, targeting knockdown of Rad18 via cholesterol-modified cy3-siRad18 delivered by engineered RGD exosomes (RGD-EXOs) synergistically inhibited the growth of OS. Our results suggest that targeting Rad18 is a promising strategy for overcoming doxorubicin resistance in human OS.

## Materials and methods

### Cell culture and treatment

The human osteosarcoma cell lines were obtained from American Type Culture Collection (ATCC) (VA, USA) and hFOB1.19, HEK293T cells were obtained from the Type Culture Collection of the Chinese Academy of Sciences (Shanghai, China). Among them, hFOB1.19 cells were cultured in DMEM/F12, 143B, HOS, MG-63 and HEK293T cells were cultured in MEM (Gibco; Thermo Fisher Scientific, Inc.), Saos-2 and U2OS cells were cultured in macoy5A(Gibco; Thermo Fisher Scientific, Inc.), all containing 10% FBS (Gibco; Thermo Fisher Scientific, Inc.) with 1% penicillin and 1% streptomycin. Cells were maintained at 37 °C in a humidified atmosphere containing 5% CO_2_.

### Genome-wide CRISPR/Cas9 screen

Human genome-wide CRISPR/Cas9 knockout library was packed into lentiviral particle and transduced into 143B cells at low multiplicity of infection (MOI). The sgRNA transduced cells were selected by puromycin. Mutant cells were cultured in vehicle and Doxorubicin for 7 days and 14 days for genetic screening. Genomic DNA was extracted from the treated cells and the sgRNA fragment was amplified. Copy number of sgRNAs was determined by high-throughput sequencing.

### Transfection and infection

SiRNAs and cholesterol-modified cy3-siRad18 were designed and synthesized by Tsingke Biotechnology Co., Ltd. (Beijing, China), and sequences of siRNAs were shown in supplementary materials. The plasmids pcDNA3.1(+)-RGD-LAMP2B-HA were constructed by our laboratory and verified by OBiO Technology Corp., Ltd. (Shanghai, China). Cells were transfected with plasmids or siRNA by Lipofectamine 3000 (Invitrogen, CA, USA) according to the manufacturer’s instructions. The Rad18 knockout was generated in OS cells using the following gRNA: Rad18 gRNA-1: TTTATCACGCGAAGAGAAGA; Rad18 gRNA-2: TAACCGCATATTAGATGAAC; Rad18 gRNA-3: TTACCAGTTCATCTAATATG.

### Analysis of cell vitality

Cell vitality was determined by cell counting kit-8 (CCK-8). The supernatant of treated cells was removed, and medium containing 10% CCK-8 was added. After 45 min incubation at 37 °C with 5% CO_2_, the absorbance at 450 nm (A450) was read using a microplate reader. The half maximal inhibitory concentration IC50 was then calculated.

### Analysis of apoptosis

Cell apoptosis rate was determined by PE Annexin V Apoptosis Detection Kit (BD Biosciences, USA). Briefly, cells were treated with indicated concentration of the drug. Then cells were harvested and stained with Annexin V-FITC and PI. Samples were analyzed using a flow cytometer (BD Biosciences, USA). Caspase-3/7 Assay Kit was used to detected the activation of Caspase-3/7.

### Colony formation

The cells were inoculated in 6-well plates, and each experimental group was inoculated with 1000 cells/well. Culture was continued for 14 days or until the number of cells in most single clones was more than 50. Medium was changed every 3 days and cell status was observed. After cloning, the cells were washed with Phosphate Buffer Saline (PBS) once, adding 2 mL 4% paraformaldehyde into each well for 30 min, and washed with PBS once. Add 1 ml crystal violet dye to each well, dye cells for 20 min. The cells were washed with PBS several times, photographed by camera.

### Comet assay

The single-cell suspension was prepared and mixed with LM agarose at 37 °C after indicated treatment. Agarose and cell mixture were coated on pretreated slides. Treated with pyrolysis solution at 4 °C. Gel electrophoresis was performed, and then the slides were neutralized. LM agarose was completely dried and stained with fluorescent dye. The degree of DNA damage was observed under a fluorescence microscope.

### RNA isolation and reverse transcription quantitative qPCR

Total RNA was isolated from cells using TRIzol® reagent (Invitrogen; Thermo Fisher Scientific, Inc.) according to the manufacturer’s protocol. Then, the RNA was reverse transcribed to cDNA with the PrimeScript™ RT Reagent kit (Takara Biotechnology Co., Ltd.). RT-qPCR was performed using SYBR-Green (Takara Biotechnology, Co., Ltd.) and an ABI Fast 7500 Sequence Detection system (Applied Biosystems; Thermo Fisher Scientific, Inc.). The gene expression level of each target gene was normalized to that of GAPDH for each sample. The primers used were provided in the supplementary materials.

### Western blotting

After the cells were harvested, the total and nuclear protein was extracted using a Nuclear and Cytoplasmic Extraction Kit (CWbiotech, China). Protein lysate was separated by SDS-PAGE and transferred to PVDF membranes (EMD Millipore), which were blocked with 5% skim milk for 1 h at room temperature. The membranes were then incubated overnight at 4 °C with antibodies as provided in the supplementary tables and further incubated with secondary antibody.

### Co-immunoprecipitation

Cells were lysed by the lysis buffer after treatment. The supernatant was mixed with antibody overnight at 4 °C, and then co-cultured with Sepharose beads for 4 h at RT. The beads were washed by washing buffer (50 mM Tris-HCl, pH 7.4, 150 mM NaCl, 0.5% NP-40) five times and the immunoprecipitates were analyzed by western blotting using the indicated antibody.

### Immunofluorescence

The cells were washed twice with cold PBS, fixed with 4% paraformaldehyde for 20 min, and permeabilized with 0.5% Triton X-100 for 30 min at room temperature. The cells were incubated overnight at 4 °C with corresponding antibodies, after being blocked for 30 min with 3% BSA. Then, the cells were incubated for 1 h with fluorescent secondary antibody. The fluorescent was photographed under a confocal laser scanning microscope using Nikon NIS-Elements software (Nikon, Tokyo, Japan).

### Histopathology

All OS tissue samples were embedded in paraffin after being fixed in 10% formalin for 24 h, and then 3 μm slices were cut. Hematoxilin-eosin (H&E) staining was performed to evaluate the morphological changes.

### Immunohistochemistry

Serial sections (4 μm) of paraffin-embedded samples were deparaffinized and rehydrated with an ethanol gradient. After the inactivation of endogenous peroxidase with 3% H_2_O_2_-methanol for 10 min, the sections were washed three times in PBS and blocked with goat serum for 20 min. Then, the sections were coated with primary antibodies, and incubated in a humid box at 4 °C overnight. After the addition of PowerVisionTM complex, tumor sections were incubated at 37 °C for 20 min, followed by DAB labeling to develop a brown color. PBS was used instead of antibodies, as a negative control.

### RNA-seq

Total RNA was extracted as described above and the RNA quality was assessed by Agilent 2100 bioanalyzer (Agilent, Santa Clara, CA). Illumina TrueSeq RNA Sample Preparation Kit V. 4.1 (Illumina Inc., San Diego, CA) was used to prepare cDNA libraries for RNA-seq. Individual barcoded libraries were analyzed using Agilent 2100 Bioanalyzer (Agilent technologies). The sequencing was performed on an Illumina Novaseq6000 by Gene Denovo Biotechnology Co. (Guangzhou, China). .

### Exosome preparation

HEK293T cell lines expressing RGD-LAMP2B were cultured in serum-free MEM for 48 h and the medium was harvested. Then the conditioned medium was centrifuged at 1000g for 15 min to remove dead cells and debris. Exosomes were extracted from supernatant by super centrifugation. All these steps were carried out at 4 °C. A BCA Protein Assay Kit (Thermo Fisher Scientific) was used to measure the total protein concentration of the exosome suspension.

### Animal studies

Male, 6-week-old BAL B/c nude mice (~ 18 g) were obtained from Gempharmatech Co., Ltd. (Nanjing, China). The Luc-143B cells (10 μ L, 1 × 10^6^ cells) were injected percutaneous into the tibia of anesthetized nude mice. The treatment was started after tumor formation on day 21. To evaluate the effect of Rad18 in vivo. The Luc-143B cells or Luc-143B with stably knockout Rad18 orthotopic transplantation nude mouse received DMSO or doxorubicin treatment (4 mg/kg *i.p.* one injection per week for 4 weeks). To evaluate targeted delivery of chemically modified siRad18 by engineered RGD-EXOs. Orthotopic mice xenografts with Luc-143B cells were divided into 5 groups with 7 mice in each group. Saline, RGD-EXOs alone, siRad18 + RGD-EXOs, doxorubicin alone and siRad18 + RGD-EXOs combined with doxorubicin (4 mg/kg *i.p.* one injection per week for 4 weeks). To examine the tumor growth, animals were administrated intraperitoneally with 2.5 mg/100uL solution of XenoLight D-luciferin (PerkinElmer, USA) and anesthetized with isoflurane for the imaging analysis. The tumor luciferase images were captured by using an IVIS 100 imaging system (PerkinElmer, USA) every 7 days. After 28 days of treatment, all nude mice were sacrificed and OS tumors were collected. The tumor weight was recorded. All animal studies were performed in accordance with protocols approved by the Ethical Committee and Institutional Review Board of Fourth Military Medical University.

### Statistical methods

All data were obtained from a minimum sample size of three per experiment and analyzed as the means ± SEM by GraphPad Prism 8.3.0 software (GraphPad Software, CA, USA) and SPSS 19.0 (SPSS, Inc., IL, USA). Two-tailed Student’s t-tests (two-sample equal variance) were adopted to test the significance of differences between two groups. Simple linear regression analysis was performed to determine the correlation between two variables. *P* < 0.05 was considered statistically significant.

## Results

### A genome-wide sgRNA library screen identified vulnerabilities in OS treated with doxorubicin

**Fig. 1:**
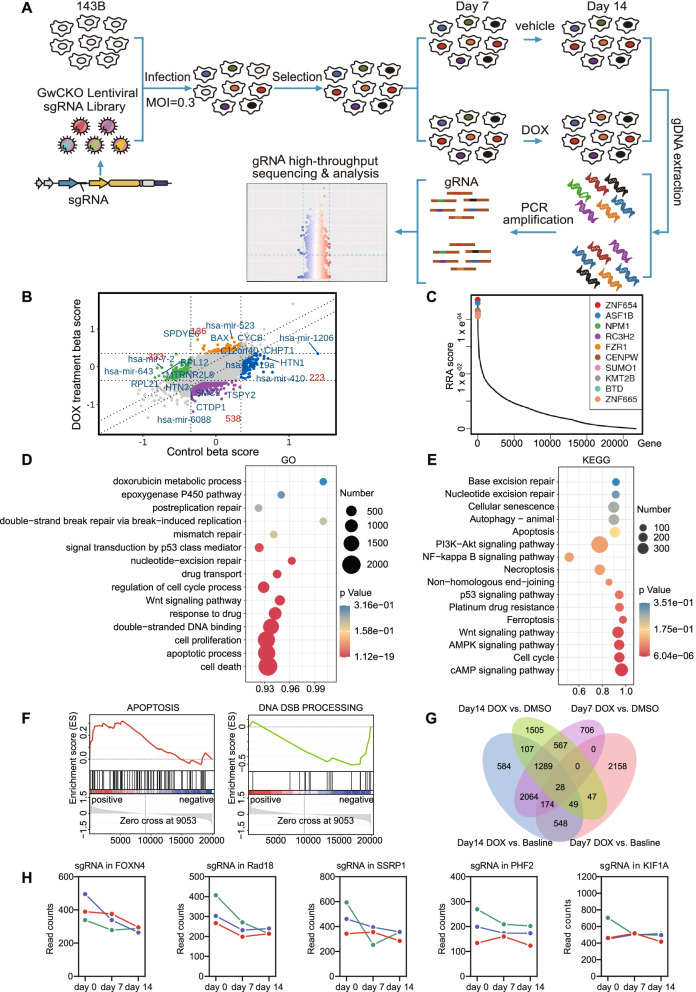
Identification of candidate regulators of OS cell resistance to Resistance. **A** Schematic diagram illustrates the workflow of genome-wide CRISPR/Cas9 knockout library screening. **B** The nine-quadrant diagram shows sgRNA sequencing results of doxorubicin treatment for 14 days. **C** SgRNA distribution of Top10 genes in negative screening. **D** GO enrichment analysis and **E** KEGG enrichment analysis of negative screening results of day 14. **F** Gene Set Enrichment Analysis (GSEA) showed that apoptosis and DNA double strand break processing pathway were influenced of negative screening on day 14. **G** Venn diagram shows differentially expressed genes in four groups described as: doxorubicin-treated group (DOX), DMSO-treated group (DMSO), none treated group (baseline). 28 genes were enriched. **H** Dynamic distribution of sgRNA Read counts of enriched genes

To functionally identify vulnerabilities of doxorubicin resistance in OS cells, we infected 143B cells with an sgRNA lentiviral library targeting 20,914 human genes, covering each gene with at least 6 independent sgRNA sequences. Cells were then cultured for 14 days under selection conditions in the presence of doxorubicin. Then, genomic DNA was extracted, and sgRNA barcodes were amplified for next-generation sequencing to identify sgRNAs and their target genes that were lost after selective culture, indicating that these genes may be critical for the maintenance of doxorubicin resistance (Fig. 1A). By analyzing the sgRNA of the control and doxorubicin treatment groups on day 14, we obtained the key genes promoting doxorubicin resistance in 143B OS cells (Fig. 1B). Among the top 10 genes, ASF1B, NPM1, FZR1, CENPW and SUMO1 were associated with the progression of multiple tumors, drug resistance and a poor prognosis in patients [18–22] (Fig. 1C), suggesting that this screening system is a reliable approach in the search for doxorubicin resistance-related targets for OS.

Through enrichment analysis of the genes obtained from negative screening, we found that the pathways about cell death, cell proliferation, cell cycle and drug transport processes, which play a key role in cell survival and chemotherapy resistance, were significantly enriched (Fig. 1D, E). In addition, gene set enrichment analysis (GSEA) showed that cell apoptosis and multiple DNA damage repair pathways were significantly affected (Fig. 1F and Supplementary Fig. 1A). We reasoned that cells with DNA damage repair defects were selected due to the DNA toxicity of doxorubicin. To further identify doxorubicin resistance-related genes, we made an analysis of data of 143B cells treated with DMSO and those treated with doxorubicin for 7 and 14 days, and revealed a total of 28 significant hits (Fig. 1G), among which genes with more than 3 independent sgRNA sequences were picked for further study (Fig. 1H and Supplementary Fig. 1B).

### Rad18 is a critical driver for doxorubicin resistance

**Fig. 2:**
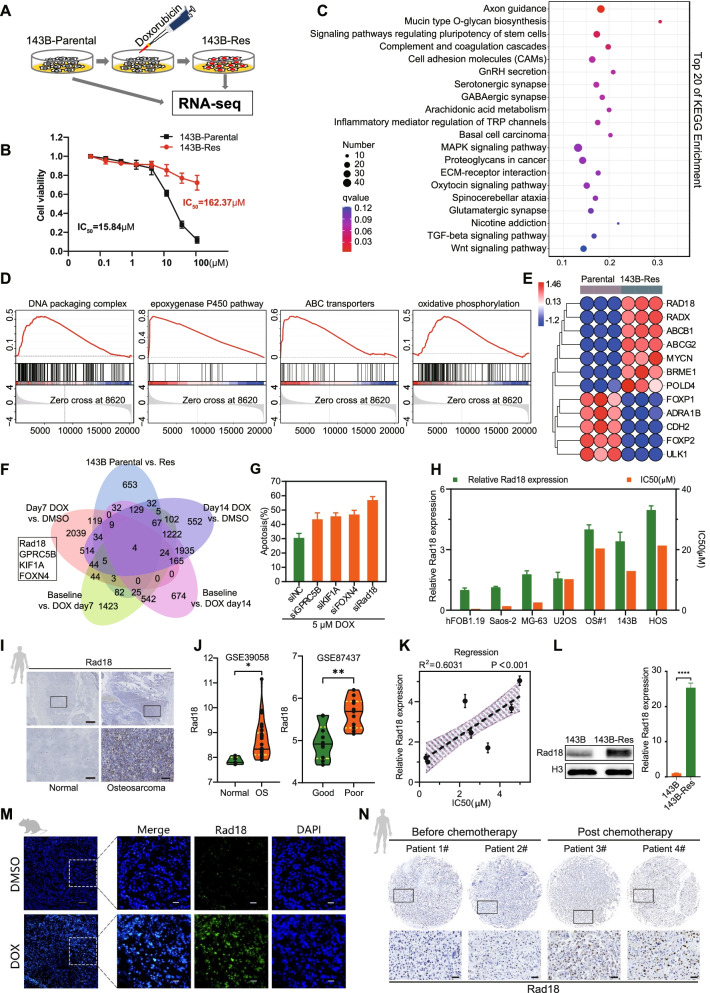
Rad18 is a determinant of doxorubicin sensitivity. **A** Induction of doxorubicin resistance 143B cells(143B-Res) from 143B-parental by doxorubicin treatment. RNA-Seq was performed in 143B-Res in parallel with 143B-parental. **B** 143B-Res and 143B-parental cells were treated with doxorubicin with the gradient concentrations. IC50was assessed by cell counting kit-8(CCK-8) at 48 h. **C** KEGG enrichment analysis of RNA-seq. Top20 signaling pathways were listed, among which a variety of drug-resistant related signaling pathways were enriched. **D** GSEA showed that DNA packaging complex, epoxygenaseP450 pathway, ABC transporters and oxidative phosphorylation pathways were significantly upregulated in 143B-Res cells when compare to the parental cells. **E** The HEAT map showed the differentially expressed genes of 143B-Res and parental cells. **F** Venn diagram showed that CRISPR/Cas9 negative screening genes were intersected with up-regulated genes of RNA-seq, and four potential drug resistance genes were obtained, which were Rad18, GPRC5B, FOXN4 and KIF1A. **G** Apoptosis rate was detected by flow cytometry in 143B cells after 5 μM doxorubicin treatment for 48 h, with which GPRC5B, KIF1A, FOXN4 and Rad18 knocked down by siRNAs. **H** IC50 and relative mRNA expression of Rad18 to doxorubicin were detected in hFOB1.19 cells and OS cell lines. **I** Representative images of immunohistochemical staining of Rad18 in normal and OS tissue sections. Scale bar =500 μm, up; Scale bar =100 μm down. **J** Online datasets analysis of the Rad18 expression in normal and OS tissue sections from GSE39058 and Rad18 expression in groups of good or poor response to chemotherapy from GSE87437. **K** Linear regression analysis of relative mRNA expression of Rad18 and IC50 in cells as mentioned above(H). **L** Protein and relative mRNA expression of Rad18 in143B-Res cells and parental. **M** Representative images of immunofluorescence staining of Rad18 on orthotopic osteosarcoma tissues of mice before and after doxorubicin treatment. Scale bar =100 μm. **N.** Representative images of IHC staining of Rad18 in OS patients before and post chemotherapy. Scale bar =50 μm

To further identify the key genes that lead to chemotherapy resistance, we constructed doxorubicin-resistant cells (143B-Res) from parental 143B cells. The successful development of doxorubicin-resistant cells was evidenced by increased cell viability under doxorubicin treatment conditions (Fig. 2A, B). RNA sequencing of doxorubicin-sensitive parental 143B cells and 143B-Res cells revealed that (Fig. 2C and Supplementary Fig. 2A), in addition to changes in cellular stress pathways, various drug-resistant pathways, including DNA replication related pathways, epoxygenase P450 pathway and ABC transporters, had dynamically shifted in 143B-Res cells (Fig. 2D and Supplementary Fig. 2B). In addition, ABCB1, ABCG2, MYCN and other known drug resistance genes were highly expressed in 143B-Res cells (Fig. 2E). These results verified the successful induction of doxorubicin resistance based on pathway changes and gene expression changes.

By overlapping the differentially expressed genes from the CRISPR/Cas9 knockout library screening and the genes with transcriptomic alterations from the RNA sequencing analysis, we obtained four potential doxorubicin resistance-related genes: Rad18, GPRC5B, KIF1A and FOXN4 (Fig. 2F). We speculated that cells with knockdown of these genes would show therapeutic sensitivity to doxorubicin. Therefore, small interfering RNAs (siRNAs) of these genes were designed for gene knockdown. The interference efficiency of each siRNA was evaluated by the relative expression of mRNA and protein (Supplementary Fig. 2C, D), and the one with the highest interference efficiency was picked for subsequent experiments. Subsequently, we found that among the four genes, Rad18 knockdown led to the most apoptosis of 143B cells induced by doxorubicin (Fig. 2G and Supplementary Fig. 2E). On the other hand, we found that Rad18 was significantly overexpressed in multiple types of cancers (Supplementary Fig. 2F) and associated with a poor prognosis of patients with sarcoma (Supplementary Fig. 2G). We further assessed the relative expression level of Rad18 in hFOB1.19 and OS cell lines and found that the expression level of Rad18 in each OS cell line was higher than that in normal cells (Fig. 2H). We also found that Rad18 was highly expressed in OS tissues compared with normal tissues according to analysis of clinical specimens and data from GSE39058 (Fig. 2I, J). Thus, we speculate that Rad18 is a determinant of doxorubicin sensitivity and the malignant progression of OS.

Subsequently, the half-maximal inhibitory concentration IC50 of doxorubicin in each cell line was measured, and regression analysis showed that the higher the expression level of Rad18 was, the stronger the tolerance of cells to doxorubicin (Fig. 2K). Doxorubicin treatment induced high Rad18 expression in vitro and in vivo (Fig. 2L, M and Supplementary Fig. 2H), which was also observed in the OS tissues of patients receiving chemotherapy (Fig. 2N). In addition, data of GSE87437 indicated the higher the expression level of Rad18 was, the worse the pathological response to chemotherapy among OS patients (Fig. 2J). Taken together, these results demonstrate that Rad18 acts as a critical player in OS cell acquisition of doxorubicin resistance.

### Rad18 depletion sensitized OS cells to doxorubicin treatment

**Fig. 3:**
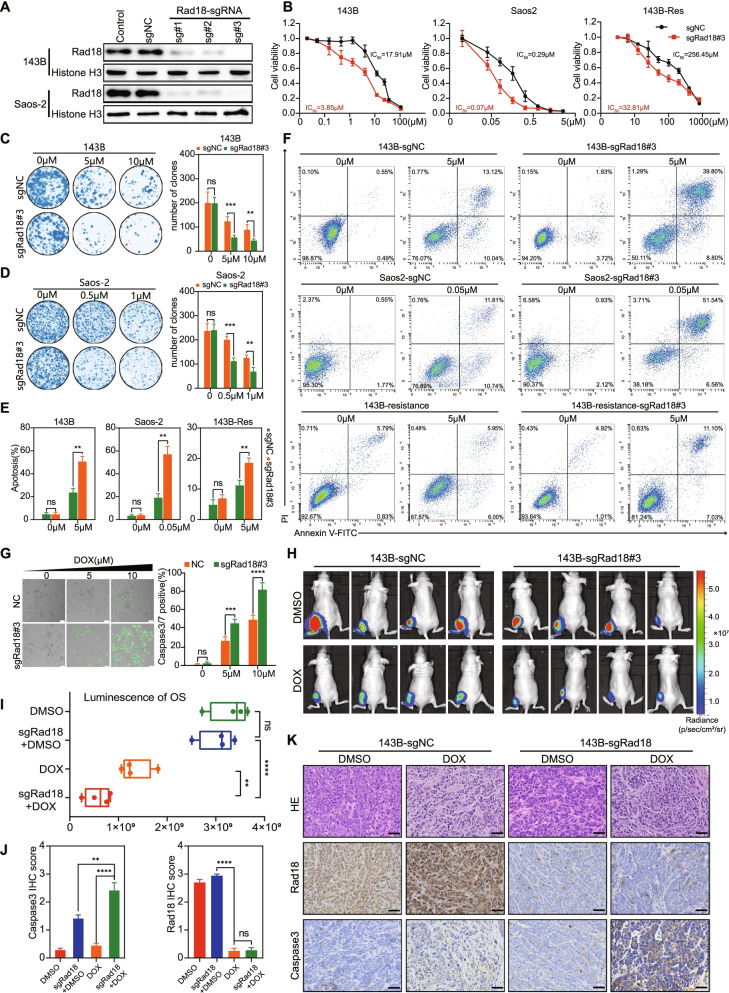
Rad18 knockout results in sensitivity to doxorubicin. **A** Rad18 protein was significantly depleted in 143B and Saos-2 cells infected with lentivirus-delivered Rad18-targeting sgRNAs. **B** Rad18 knock-out cells exhibited increased sensitivity to doxorubicin, as assessed by cell viability assay under doxorubicin treatment for 48 h at indicated doses. **C** and **D** Representative images and analysis of cell densities in WT and Rad18 knock-out cells treated with doxorubicin or vehicle and stained with crystal violet after 14 days. **E** and **F** Knockdown of Rad18 augmented doxorubicin induced apoptosis in OS cells **G** Caspase-3/7 activity apoptosis assay in WT and Rad18 knock-out cells treated with doxorubicin. Scale bar =25 μm. **H** Representative bioluminescent images of nude mice xenografted with WT or Rad18 knock-out 143B-luc cells, which were treated with doxorubicin for 4 weeks (4 mg/kg, intraperitoneal (*i.p.*) injection, once a week). **I** Quantification of the bioluminescence signal in mice received doxorubicin described as above(H). **J** Representative images of HE and IHC staining of OS tissue sections from aforementioned indicated groups. Scale bar =50 μm. **K** Quantification analysis of cleaved caspase-3 and Rad18 IHC staining from aforementioned indicated groups

To investigate the effects of Rad18 on doxorubicin resistance, we generated stable Rad18 knockout subclones in 143B and Saos-2 cells by CRISPR–Cas9 lentiviral vectors with sgRNA#3 of Rad18 (Fig. 3A). We found that Rad18 knockout increased the sensitivity to doxorubicin in 143B, Saos-2 and 143B-Res cells (Fig. 3B), We further overexpressed Rad18 in 143B and Saos-2 cell lines (Supplementary Fig. 3A). while overexpression of Rad18 led to doxorubicin resistance (Supplementary Fig. 3B). Rad18-knockout subclones showed no significant effects on OS cell proliferation in vitro, whereas knockout of Rad18 significantly induced a reduction in the number of clones after doxorubicin treatment (Fig. 3C, D). In addition, overexpression of Rad18 significantly restored the reduction in the number of clones caused by doxorubicin (Supplementary Fig. 3C, D). Thus, Rad18 expression is closely related to doxorubicin resistance. Furthermore, we found that doxorubicin-induced apoptosis significantly increased in all three Rad18-knockout cell lines (Fig. 3E, F). Subsequently, a caspase-3/7 staining assay revealed that doxorubicin induced increased apoptosis in the Rad18-knockout group (Fig. 3G). In contrast, doxorubicin-induced apoptosis and caspase-3/7 activation were alleviated after overexpression of Rad18 (Supplementary Fig. 3E, F). These suggest that cytotoxicity of doxorubicin was correlated with expression level Rad18.

Next, we sought to confirm the phenomenon in vivo. 143B and Rad18 knockout 143B cells carrying luciferase were injected into the tibia of nude mice, and orthotopic OS tumors were formed after 3 weeks. After treatment with doxorubicin or DMSO for 4 weeks, OS was significantly inhibited by doxorubicin, and the inhibition was significantly increased after Rad18-knockout (Fig. 3H, I). HE staining and immunohistochemical staining indicated that doxorubicin caused more OS necrosis and caspase-3 activation in the Rad18-knockout group than in the control group, suggesting that Rad18 knockout can increase doxorubicin-induced apoptosis (Fig. 3J, K). Together, these data demonstrate that knockout Rad18 induces OS sensitive to doxorubicin treatment both in vivo and in vitro.

### Rad18 promotes the HR repair pathway to increase the tolerance of doxorubicin-induced DNA damage

**Fig. 4:**
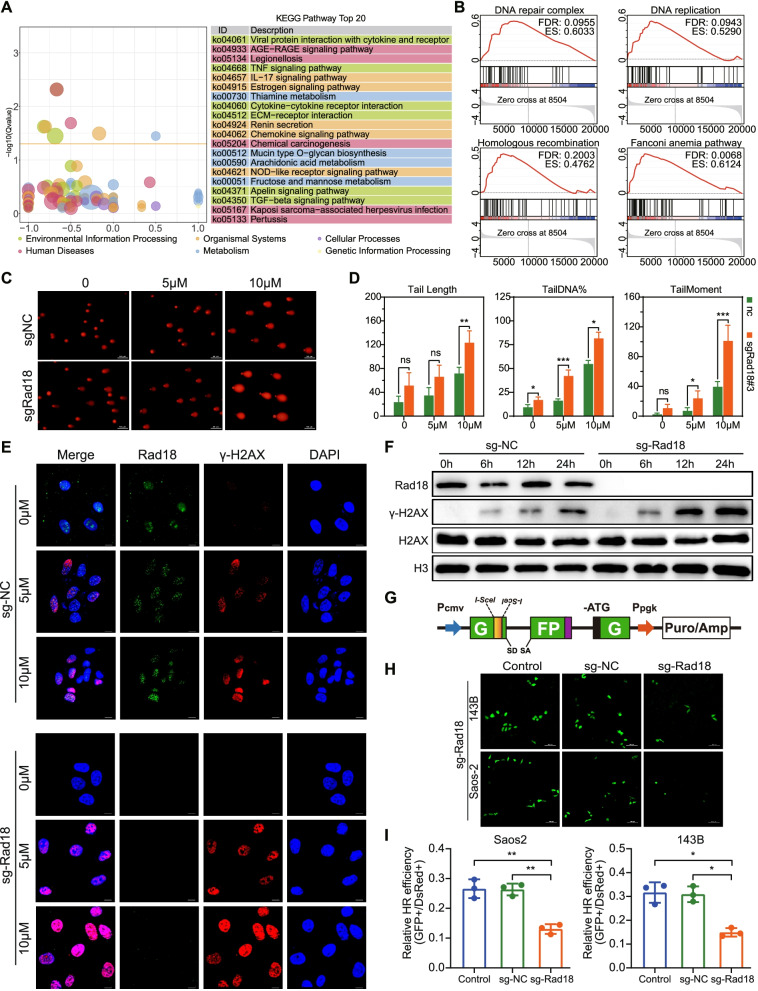
Rad18 knockout reduces the efficiency of HR-mediated DNA damage repair. **A** KEGG enrichment analysis and **B** GSEA analysis of WT and Rad18 knock-out 143B cells. **C** Representative images of the comet assay in WT and Rad18 knock-out 143B cells. Scale bar =50 μm. **D** Quantification of TailDNA%, TailMoment and Tail length of the described comet assay(C). **E** Immunofluorescence staining of WT and Rad18 knock-out 143B cells with γ-H2AX and Rad18 antibodies. Scale bar =10 μm. **F** Western blot analysis of γ-H2AX and Rad18 in WT and Rad18 knock-out 143B cells with 5 μM doxorubicin treatment for 0 h, 6 h, 12 h and 24 h. **G** Diagram of HR reporter construction. **H** and **I** Representative fluorescent images of OS cells transfected with HR reporter, and relative HR efficiencies were calculated. Scale bar =100 μm

To determine how Rad18 functions, transcriptomic sequencing was performed on 143B and Rad18-knockout 143B cells. DNA replication and DNA repair pathways, such as the HR and Fanconi anemia pathways, were significantly enriched (Fig. 4A, B and Supplementary Fig. 4A). Therefore, we characterized doxorubicin-induced DNA damage by a comet assay to determine whether Rad18 regulates DNA repair. The results showed that the DNA comet tail moment and tail length in Rad18-knockout cells were extended compared to those in control cells; and these trends were reversed in Rad18-overexpressing cells, suggesting that Rad18 is a key factor in the tolerance of doxorubicin-induced DNA damage (Fig. 4C, D and Supplementary Fig. 4B, C).

Since DSBs are serious consequence of DNA damage caused by doxorubicin, we investigated whether Rad18 regulates DSBs by assessing the foci of γ-H2AX, which is phosphorylated in response to DSBs [23]. We found that Rad18-knockout cells contained a greater number of γ-H2AX-positive foci, and the difference remained significant at 24 h, suggesting that Rad18-knockout cells were unable to efficiently repair DSBs (Fig. 4E). The enhanced accumulation of γ-H2AX in Rad18-knockout cells was further confirmed by western blotting (Fig. 4F). In Rad18-overexpressing cells, H2AX phosphorylation was weakened, suggesting that Rad18 could reduce the accumulation of DSBs (Supplementary Fig. 4D, E).

HR is one of the key pathways to repair DSBs [24]. Our GSEA also showed that the HR pathway was enriched after Rad18 knockout (Fig. 4B), so we hypothesized that Rad18 regulates HR to influence the repair of doxorubicin-induced DSBs. Subsequently, we employed HR-GFP, the DSB repair reporter that allows quantification of the activities of HR. Fluorescence microscopy showed that Rad18 knockout reduced HR-mediated DSB repair efficacy in both Saos-2 and 143B cells treated with doxorubicin, suggesting that Rad18 knockout inhibits DSB repair through inhibition of the HR pathway (Fig. 4G-I). Collectively, we found HR deficiency in Rad18 knockout cells. This deficiency resulted in a delay in DNA damage repair, which aggravated doxorubicin-mediated DNA damage and enhanced doxorubicin cytotoxicity.

### Rad18 interacts with MRE11 and enhances MRN complex formation

**Fig. 5:**
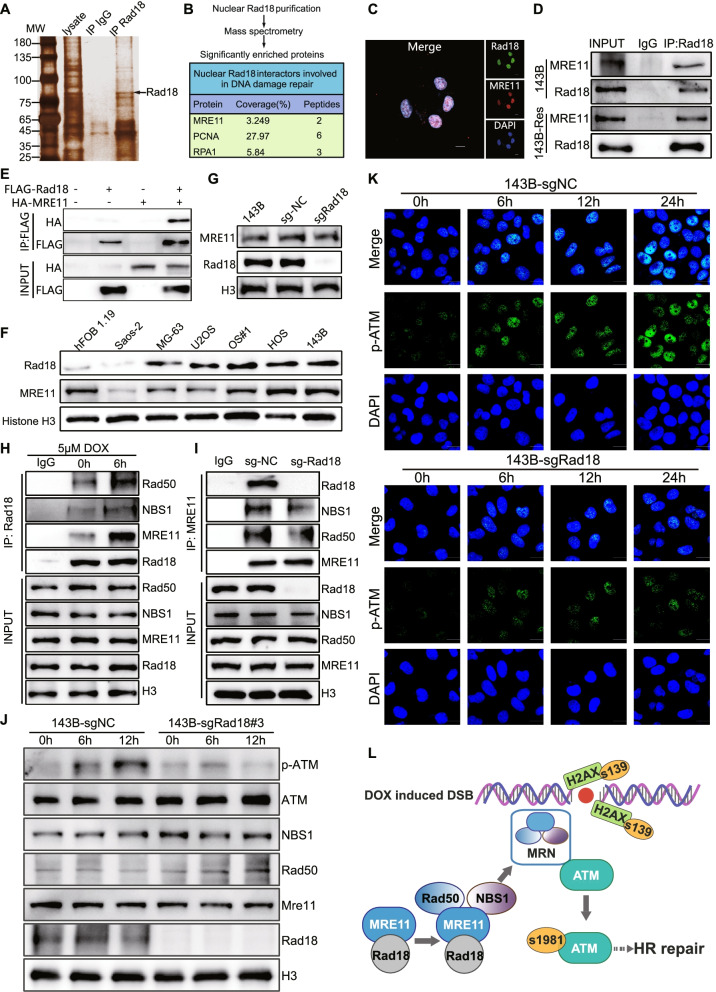
Rad18 interact with MRE11 and promote formation of MRN complex. **A** SDS-PAGE separation and silver staining of proteins co-immunoprecipitated with a Rad18-specific antibody. Interacting proteins were subjected to mass spectrometry analysis. **B** List of MRE11, PCNA and RPA1 picked from interactors of Rad18. **C** Representative IF images of double staining MRE11 and Rad18. Scale bar =10 μm. **D** Co-IP showed that MRE11was pulled down by Rad18. **E** Co-IP showed that HA-tagged MRE11 was pulled down by Flag specific antibody in lysate of 293 T cells which co-transfected with FLAG-tagged Rad18 and HA-tagged MRE11 expression vectors. **F** and **G** Protein expression of Rad18 and MRE11in hFOB1.19 and OS cell lines. **H** Doxorubicin treatment increased interaction between Rad18 and MRE11. Total lysates derived from 143B cells treated with 0 μM or 5 μM doxorubicin were immunoprecipitated with Rad18 antibodies. The precipitates were probed with the indicated antibodies. **I** Rad18 knockout decreased MRN complex formation. Total lysates derived from 143B cells treated with 5 μM doxorubicin for 0 h and 6 h were immunoprecipitated with Rad18 antibodies. The precipitates were probed with the indicated antibodies. **J** Western-blotting analysis showed Rad18 knockout inhibited doxorubicin induced ATM phosphorylation. **K** Immunofluorescence staining of 143B-sgNC and 143B-sgRad18 cells with p-ATM antibodies after the cells were infected with lenti-sgNC or lenti-sgRad18 and subsequently treated with doxorubicin in time gradient. Scale bar =10 μm. **L** Schematic diagram of the mechanism that Rad18 promotes HR pathway through interaction with MRE11

To gain further insights into the mechanism of Rad18, 143B cells treated with 5 μM doxorubicin for 6 h were collected and lysed, and e immunoprecipitated with an anti-Rad18 antibody. Co-IP proteins were separated using SDS-PAGE (Fig. 5A). The target strip was excavated and for mass spectroscopy (MS). We focused on the proteins related to DNA damage repair, and MRE11, PCNA and RPA were identified. (Supplementary Fig. 5A). Previous studies have shown that PCNA and RPA are definite interacting proteins of Rad18, but neither of them is directly involved in HR. [25, 26] MRE11, a DNA DSB repair protein, is a key initiation of the HR pathway, while the interaction between Rad18 and MRE11 protein has not been elucidated. The phosphatase domain of MRE11 has single-stranded DNA endonuclease and double-stranded DNA exonuclease activities, which are mainly responsible for pruning the ends of DNA broken strands and subsequently initiating the DSB repair pathway [27, 28]. We innovatively found that MRE11 might be a binding partner of Rad18 by IP/mass spectrometry (Fig. 5B and Supplementary Fig. 5A). In addition, we predicted the relationship between Rad18 and MRE11 and HR repair pathways through the Search Tool for the Retrieval of Interacting Genes/Proteins (STRING) database (Supplementary Fig. 5B). Therefore, we speculated that Rad18 may regulate the HR pathway by interacting with MRE11, thereby affecting DNA damage repair (DDR).

To explore the above hypothesis, we first explored the interaction between Rad18 and MRE11. After immunofluorescence staining, we found the colocalization of Rad18 and MRE11 through regression analysis of fluorescence intensity (Fig. 5C, Supplementary Fig. 5C, D). Moreover, an immunoprecipitation (IP) assay of Rad18/MRE11 was performed in 143B and 143B-RES cells. MRE11 was detected from IP products of Rad18, suggesting that Rad18 could interact with MRE11 (Fig. 5D). To further verify the interaction, HEK293T cells were co-transfected with flag-tagged Rad18 and HA-tagged MRE11 expression vectors. Protein complexes were immunoprecipitated with an anti-FLAG antibody. The lysates were examined by western blot using anti-Flag and anti-HA antibodies. Analysis under specific loading conditions revealed that Rad18 interacts with MRE11 (Fig. 5E).

Based on the above conclusions, we then explored whether Rad18 regulates the expression level of MRE11 to affect the HR pathway. The expression levels of MRE11 in OS cell lines showed different trends from Rad18 (Fig. 5F). In addition, the expression level of MRE11 was not changed after knockout or overexpression of Rad18, indicating that Rad18 does not regulate the expression of MRE11 (Fig. 5G, Supplementary Fig. 5E). Considering that MRE11 is the key active region of the MRN (MRE11/RAD50/NBS1) complex, we further studied the influence of Rad18 on the MRN complex [27]. We first observed the enhanced interaction between Rad18 and the MRN complex after treated with 5 μm doxorubicin for 6 h (Fig. 5H). Additionally, the formation of the MRN complex was disrupted after Rad18 knockout, suggesting that Rad18 could promote the formation of the MRN complex (Fig. 5I, Supplementary Fig. 5F). However, we found that regardless of whether Rad18 was knocked out or overexpressed, the expression levels of NBS1, MRE11 and RAD50 did not change significantly (Fig. 5J, Supplementary Fig. 5G). This means that the effect of Rad18 might be promoting the formation of the MRN complex as a whole rather than the expression of the individual components. The MRN complex promotes ATM activation by inducing its autophosphorylation at S1981 [29]. Activated ATM rapidly phosphorylates a large number of substrates in local chromatin, providing scaffolding for assembly of higher-order complexes to repair damaged DNA, which facilitates the HR pathway [29, 30]. Therefore, we detected the phosphorylation level of ATM as a marker of HR activation to reveal the functional status of MRN complexes. We found that ATM phosphorylation was significantly inhibited when Rad18 was knocked out, and it was increased after Rad18 was overexpressed (Fig. 5J, Supplementary Fig. 5G). Immunofluorescence yielded consistent results, indicating that Rad18 is involved in the regulation of ATM phosphorylation (Fig. 5K, Supplementary Fig. 5H). Collectively, we verified that Rad18 interacts with MRE11 to promote the formation of the MRN complex, which facilitates the activation of ATM to promote the HR pathway (Fig. 5L).

### Targeted delivery of chemically modified siRad18 by engineered RGD-EXOs sensitized OS cells to doxorubicin treatment

**Fig. 6:**
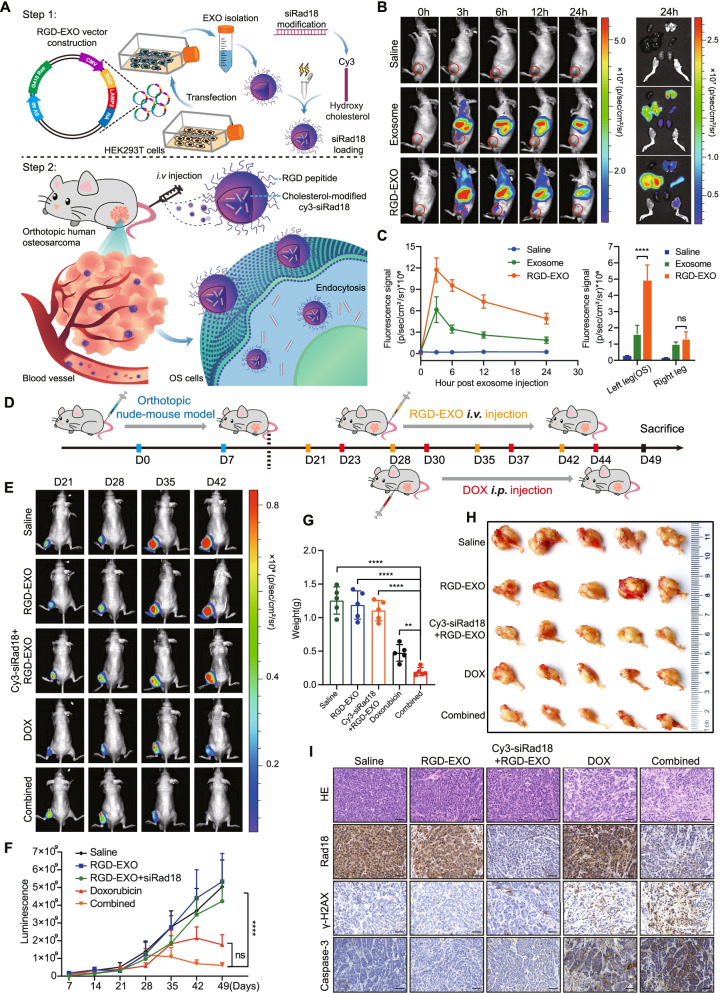
Targeted knockdown of Rad18 based on exosome delivery and RNAi technology increases the sensitivity of osteosarcoma cells to doxorubicin. **A** Schematic image. Process of RGD-EXO construction, isolation, siRad18 loading, animal tail vein injection. **B** Left, NIR fluorescence imaging of mice at 0 h, 3 h, 6 h, 12 h, and 24 h post-injection with DID-stained RGD-EXOs or Exosome, which loaded cholesterol-cy3-siRad18. Right, biodistribution of DID-stained RGD-EXOs and Exosome were tracked 24 h after IV injection. **C** Quantitative analysis of NIR fluorescence signal from nude mice that received cholesterol-cy3-siRad18 loaded and DID stained RGD-EXOs and Exosome. **D** Time line of nude mice receiving combination therapy. **E** and **F** Representative images and data analysis of animal bioluminescence at different time points in each treatment group. **G** and **H** Weight and appearance of OS in vitro after the last treatment. **I** Representative images of H&E and Rad18, γ-H2AX and cleaved Caspase-3 IHC in OS sections of indicated groups. Scale bar =50 μm

To further improve the efficiency of chemotherapy in vivo, we designed a treatment regimen of Rad18 knockdown combined with doxorubicin chemotherapy. Targeted delivery of siRNA loaded in engineered exosomes has become our first choice [31]. Chemical modification effectively improves the stability of siRNA in blood, but the lack of targeting limits its application. Exosomes have become the focus of drug delivery research, which are characterized by high biosafety, easy preparation, and weak immunogenicity [32, 33]. Previously successful applications of engineered exosomes for the targeted delivery of siRNAs guided our approach [34–36]. In addition, RGD-EXOs have been proven to target OS in vivo [37–39]. Therefore, we designed a targeted delivery scheme based on engineered RGD-EXOs and chemically modified siRNA (Fig. 6A). We constructed a fusion expression vector of RGD and LAMP2, and LAMP2 carried the integrin-targeting RGD peptide to exosome membrane surface, allowing exosomes to target osteosarcoma cells with high integrin expression. We designed siRad18 to knock down the expression of Rad18 in osteosarcoma tissues. SiRad18 was modified with cholesterol to increase stability and coupled with cy3 groups for tracer. SiRNA was loaded into RGD-EXOs by electrical transfer. After loading, RGD-EXOs solution was injected through the tail vein and delivered siRad18 to OS cells of orthotopic osteosarcoma.

We investigated the efficiency of cholesterol-modified cy3-siRad18 delivered to OS cells by engineered exosomes. First, through electron microscopy, we found that RGD-EXOs loaded with siRad18 and empty RGD-EXOs had no obvious morphological changes and little difference in particle size (Supplementary Fig. 6A). Second, siRad18 could be more efficiently integrated into cells via RGD-EXOs than via transfection alone to facilitate gene knockdown (Supplementary Fig. 6B). Finally, cholesterol-modified cy3-siRad18 delivered by engineered exosomes reduced the mRNA and protein expression of Rad18 (Supplementary Fig. 6B, C). In addition, Rad18 knockdown cells had significantly higher sensitivity to doxorubicin (Supplementary Fig. 6D). These results suggest that cholesterol-modified siRNA delivered by engineered RGD-EXOs can function in vitro.

Distribution detection in vivo showed that cholesterol-modified cy3-siRad18 loaded by RGD-EXOs were widely distributed in the liver and spleen but less in the heart, lung and kidney, which may be related to the rich capillary networks in the liver and spleen. Compared with ordinary exosomes, RGD-EXOs were effectively transported to the site of OS (the tibia) and released siRad18, knocking down Rad18 in OS, suggesting that cholesterol-modified cy3-siRad18 loaded by RGD-EXOs can effectively play the role of targeted delivery and knockdown in vivo (Fig. 6B, C and supplementary Fig. 6E). Moreover, we verified the biosafety of RGD-EXOs and RGD-EXOs-siRad18 in vivo. HE-stained sections of all organs showed no tissue or organ damage, and liver and kidney function analysis showed that control RGD-EXOs and RGD-EXOs-siRad18 had no significant effect on the liver and kidney function of nude mice (Supplementary Fig. 6F, G). Subsequently, we applied a combined treatment in which cholesterol-modified cy3-siRad18 loaded by RGD-EXOs were injected through the tail vein and doxorubicin chemotherapy was injected intraperitoneally to inhibit orthotopic OS in nude mice. The treatment lasted for 4 weeks; RGD-EXOs were injected through the tail vein 2 days earlier than doxorubicin to take full advantage of the effects of Rad18 knockdown (Fig. 6D).

In vivo bioluminescence experiments showed that OS tumor growth in the doxorubicin group and the combined treatment group was significantly inhibited compared with that in the no doxorubicin treatment group. It is worth mentioning that although the tumor volume in the combined treatment group was lower than that in the doxorubicin group, there was no significant difference between the two groups (Fig. 6E, F). However, the tumor weight of the combined treatment group was significantly lower than that of the doxorubicin group (Fig. 6G, H). This may be caused by the inadequate sensitivity of bioluminescence signal measurement and the large difference in signal intensity within the group. These results indicated that cholesterol-modified cy3-siRad18 loaded by RGD-EXOs combined with doxorubicin enhanced the in vivo lethality of doxorubicin against OS. Immunohistochemical staining indicated that engineered RGD-EXOs delivered cholesterol-modified cy3-siRad18 could effectively inhibit the expression of Rad18 in OS. The phosphorylation level of H2AX and the expression level of cleaved caspase-3 in the combined treatment group were significantly higher than those in the doxorubicin group (Fig. 6I). This suggests that RGD-EXOs mediated targeted knockdown of Rad18 aggravated doxorubicin-induced DNA damage and promoted OS apoptosis.

## Discussion

Doxorubicin exerts cytotoxic effects by inhibiting DNA replication, leading to DNA damage and even DSBs [40, 41]. Homologous recombination (HR) repair uses undamaged sister chromatids as homologous templates to restore DNA synthesis and rescue DSBs and other severe DNA damage, thus inducing resistance of OS cells to chemotherapy [42, 43]. Here, we found several key genes that mediated doxorubicin resistance in OS cells by genome-wide CRISPR screening combined with transcriptomic sequencing, including Rad18, KIF1A, FOXN4 and GPRC5B. Among the four candidate genes, Rad18 was the most significant key gene in doxorubicin sensitivity to OS cells. Analysis of clinical samples and datasets showed that Rad18 related to occurrence and poor chemotherapy response of OS. Then, we conducted in vitro and in vivo experiments and confirmed that Rad18 knockout could significantly increase the toxicity of doxorubicin to OS.

We further explored the mechanism of Rad18 and found that with Rad18 knockout, the HR repair pathway was significantly inhibited. Previous studies have shown that Rad18, an E3 ubiquitin ligase, is one of the key proteins in DNA damage repair, but most studies have been limited to the role of Rad18 in the translesion DNA synthesis (TLS) pathway [14, 44, 45]. It should be mentioned that doxorubicin can target topoisomerase and ultimately lead to severe DNA damage, such as DNA DSBs [46]. However, TLS is involved in single-strand DNA damage pathways, such as blocked replication forks, which cannot deal with DSBs [47]. Therefore, we believe that TLS may not be involved in the DNA damage repair mechanism caused by doxorubicin, which is consistent with the existing rare research related to doxorubicin and TLS. Similar to ours, previous studies have observed that Rad18 can promote DSB repair and regulate HR, but the specific mechanisms and targets need further research [48–52].

Therefore, IP-MS was adopted to further explore the downstream molecules of Rad18, and it was found that Rad18 colocalized with MRE11 and could directly interact with MRE11. Interestingly, we found that Rad18 didn't regulate the expression level of MRE11, so we hypothesized that Rad18 could affect the biological function of MRE11. MRE11 can form ternary complexes with RAD50 and NBS1, and MRN complexes bind to DNA to prune the ends of DNA broken chains and then initiate the DBS repair pathway [53, 54]. Since the function of MRE11 in the HR pathway depends on the integrity of the MRN complex, we further assessed the influence of Rad18 on the formation of the MRN complex [55]. The results showed that Rad18 could interact with MRE11 and further promote ATM phosphorylation by facilitating the formation of the MRN complex, mediate the cascade amplification of the phosphorylation activation pathway, and thus promoting DNA damage repair. However, the specific mechanism by which Rad18 regulates MRE11 remains a puzzle. On the basis of previous studies, we speculate that there are two possible different mechanisms. On the one hand, Rad18 can associate with K63-linked Polyubiquitylated Chromatin proteins through the UBZ domain [49, 56]. Therefore, Rad18 may promote the recruitment and assembly of MRN complexes at fragile DNA through a k63-ubiquitination dependent way. On the other hand, a molecular sponge protein, C1QBP, was found to stabilize MRE11 and regulate the assembly of MRN complexes [53], suggesting Rad18 could also function as a molecular sponge. In any case, further work is needed.

In clinical practice, the establishment of biological indicators of chemotherapy response to guide the selection of chemotherapy regimen can avoid excessive chemotherapy and ensure a reliable clinical benefit for patients. Here, we showed that high expression of Rad18 in OS cells correlated with a doxorubicin resistance, whereas patients with high Rad18 expression exhibited a poor prognosis. Therefore, we recommend evaluating the expression of Rad18 in OS tumors to select patients who may respond better to doxorubicin. Our study revealed that depletion of Rad18 largely abrogates doxorubicin resistance in OS, which restores the chemosensitivity of resistant OS. Thus, we propose that Rad18 can be a target for OS to enhance chemotherapy sensitivity, which decrease the dose of systemic medication and reduce the systemic damage of doxorubicin. In addition, we attempt to improve siRNA knockdown efficiency in a variety of ways and find or design stable and specific novel inhibitors of Rad18 in future work, which will further improve clinical efficacy and reduce systemic toxicity of doxorubicin.

## Conclusions

Overall, we identified Rad18 as a key factor leading to OS resistance, and innovatively found that Rad18 could interact with MRE11 and promote the formation of the MRN complex, which then promoted DNA damage repair mediated by the HR pathway. The therapeutic effect of doxorubicin in OS could be significantly enhanced by Rad18 knockout. In addition, we explored the targeted delivery of chemically modified siRad18 by engineered RGD-EXOs, which might be a potential strategy for adjuvant therapy along with chemotherapy for patients with OS.

## Supplementary Information

**Additional file 1.**

**Additional file 2.**

## Data Availability

The datasets used and/or analyzed during the current study are available from the corresponding author and RNA sequencing data have been deposited to SRA platform (https://www.ncbi.nlm.nih.gov/sra) and are available under accession number: PRJNA809562 and PRJNA809642.

## Abbreviations

OS: Osteosarcoma
Rad18: E3 ubiquitin-protein ligase Rad18
MRE11: Meiotic recombination 11
HR: Homologous recombination
MRN: MRE11-RAD50-NBS1
ACTT: American Type Culture Collection
MOI: Multiplicity of infection
CCK-8: Cell counting kit-8
PBS: Phosphate Buffer Saline
GSEA: Gene set enrichment analysis
siRNA: Small interfering RNA
IP: Immunoprecipitation
MS: Mass spectrometry
STRING: Search Tool for the Retrieval of Interacting Genes/Proteins
DDR: DNA damage repair
TLS: Translesion DNA synthesis
qPCR: Quantitative PCR
