# Supplementary material for: Genome-wide CRISPR screen identified Rad18 as a determinant of doxorubicin sensitivity in osteosarcoma
Source: J Exp Clin Cancer Res. 2022 Apr 23;41:154. doi: 10.1186/s13046-022-02344-y (PMC9034549; doi:10.1186/s13046-022-02344-y)
Supplement: Supplementary file 2 — Additional file 2. [file 13046_2022_2344_MOESM2_ESM.docx]

**Supplementary Materials**

**Title:** **CRISPR screen identified Rad18 as a determinant of doxorubicin sensitivity in osteosarcoma**

Mingrui Du^1,2*^, Jintao Gu^2*^, Chenlin Liu^2*^, Nannan Liu^3*^, Zhe Yu^1^, Chengpei Zhou^1^, Wei Heng^1^, Zhengcong Cao^2^, Feilong Wei^1^, Kailong Zhu^1^, Yingwen Wang^4^, Wei Zhang^2^, Xiaochang Xue^5,6#^, Yong Zhang^1#^, Jixian Qian^1#^

^1^Department of Orthopedics, The Second Affiliated Hospital, The Fourth Military Medical University, Xi'an, China

^2^State Key Laboratory of Cancer Biology, Biotechnology Center, School of Pharmacy, The Fourth Military Medical University, Xi’an, China

^3^Experimental Teaching Center of Basic Medicine, The Fourth Military Medical University, Xi'an, China

^4^Preclinical School of Medicine, The Fourth Military Medical University, Xi’an, China

^5^National Engineering Laboratory for Resource Development of Endangered Crude Drugs in Northwest China, College of Life Sciences, Shaanxi Normal University, Xi’an, China

^6^The Key Laboratory of Medicinal Resources and Natural Pharmaceutical Chemistry (Shaanxi Normal University), The Ministry of Education, College of Life Sciences, Xi’an, China

^*^These authors contributed equally to this work.

^#^Corresponding authors:

**Supplementary Figures**


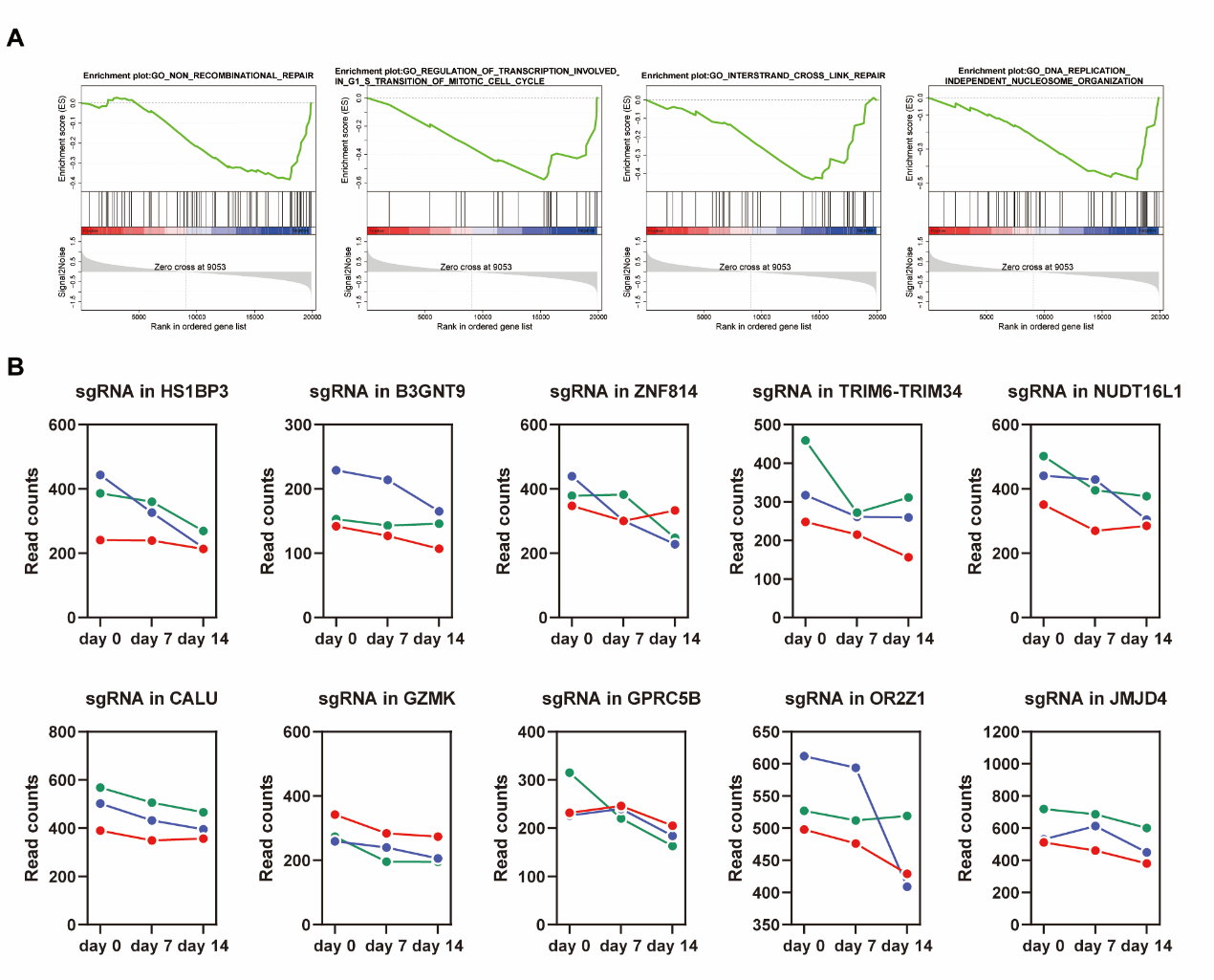


**Supplementary Fig. 1 (Related to Fig. 1).**

**A.** GSEA analysis on the genes obtained from negative screening. **B.** sgRNA read counts of some selected gene which may be potentially critical for the maintenance of doxorubicin resistance


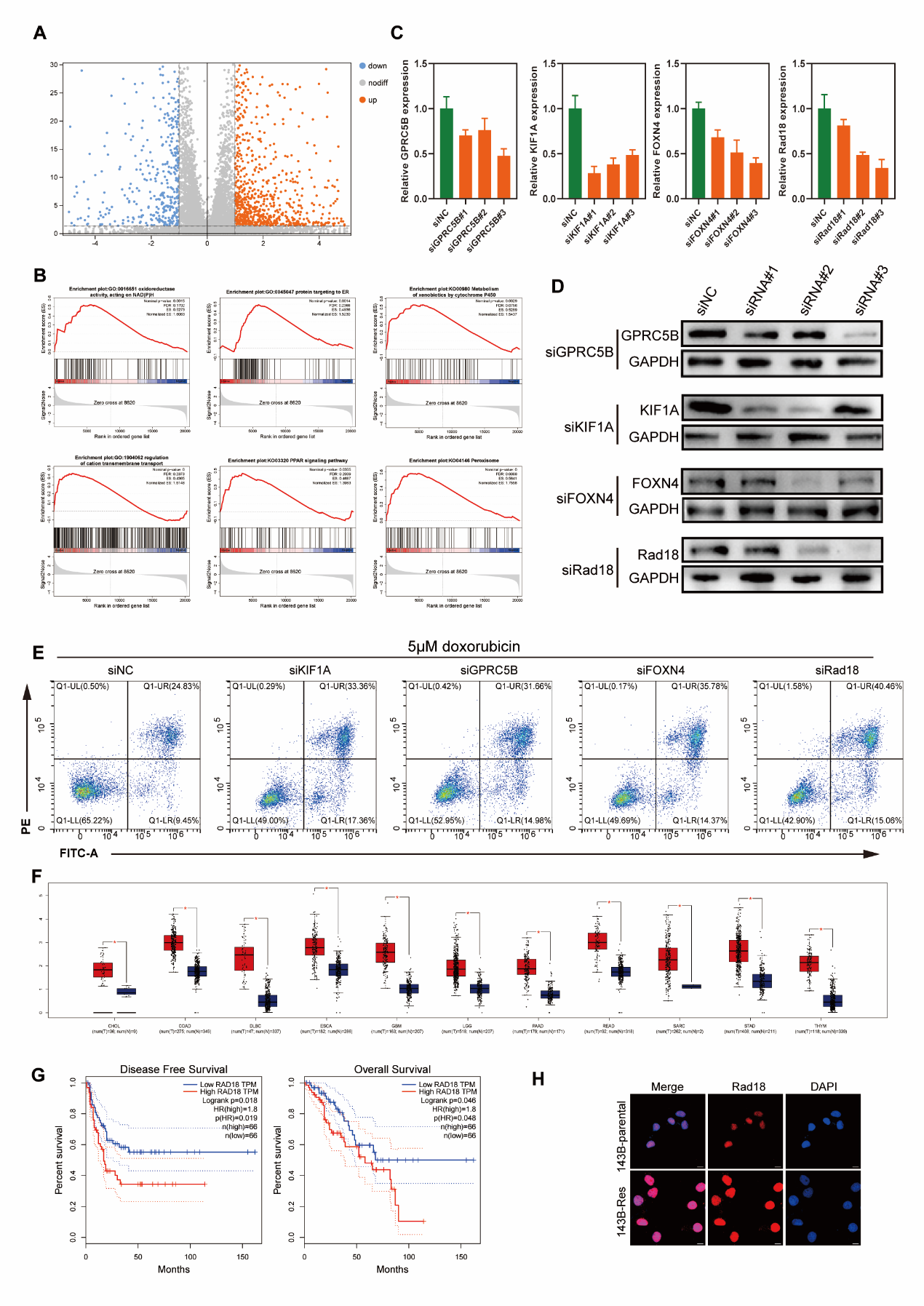


**Supplementary Fig. 2 (Related to Fig. 2)**

**A.** Volcanic map analysis of RNA-seq about 143B-parental and 143B-Res. **B.** GSEA analysis of RNA-seq about 143B-parental and 143B-Res. **C. and D.** The interference efficiency of Rad18, GPRC5B, KIF1A and FOXN4 were evaluated by the relative expression of mRNA and protein. **E.** After gene knockdown the four candidate genes, apoptosis rate in 143B cells treated with 5 μM doxorubicin for 48h was detected by cytometry respectively. **F.** Expression level of Rad18 in in multi-type cancers. **G.** Survival analysis between patients with high Rad18 and low Rad18 in sarcoma. **H.** Higher expression of Rad18 in 143B-res-cells. Scale bar: 10 µm.


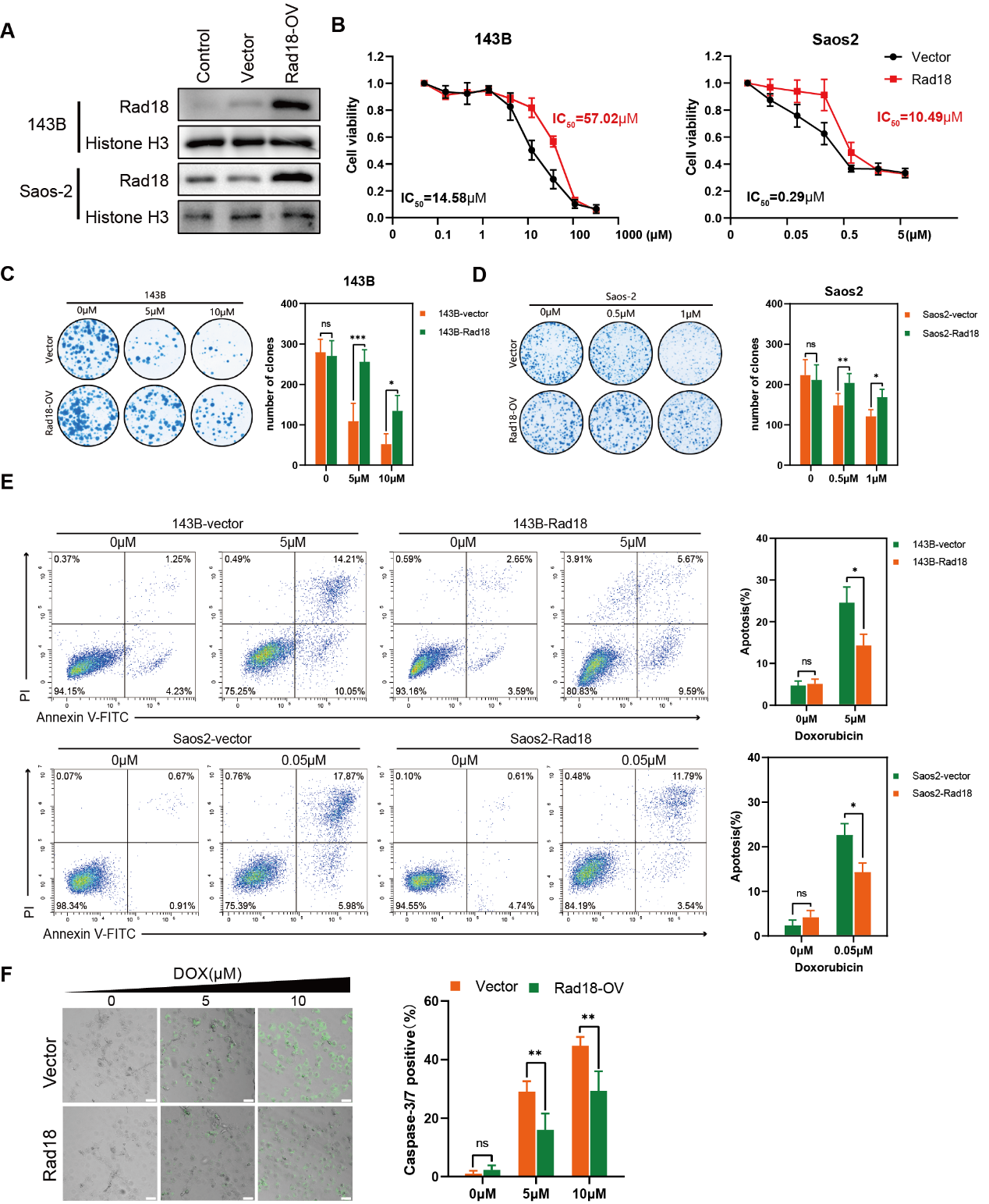


**Supplementary Fig. 3 (Related to Fig. 3)**

**A.** Construction of overexpressed Rad18 in 143B and Saos2 cell lines. **B.** Changes of cell viability in 143B and Saos2 cell lines upon doxorubicin treatment after overexpression of Rad18. **C.** Change in number of clones in 143B cells upon doxorubicin treatment after overexpression of Rad18. **D.** Change in number of clones in Saos2 cells upon doxorubicin treatment after overexpression of Rad18. **E.** Change of apoptosis rate in Saos2 cells upon doxorubicin treatment after overexpression of Rad18. **F.** Change of caspase-3/7 activation in 143B cells upon doxorubicin treatment after overexpression of Rad18. Scale bar: 25 µm.


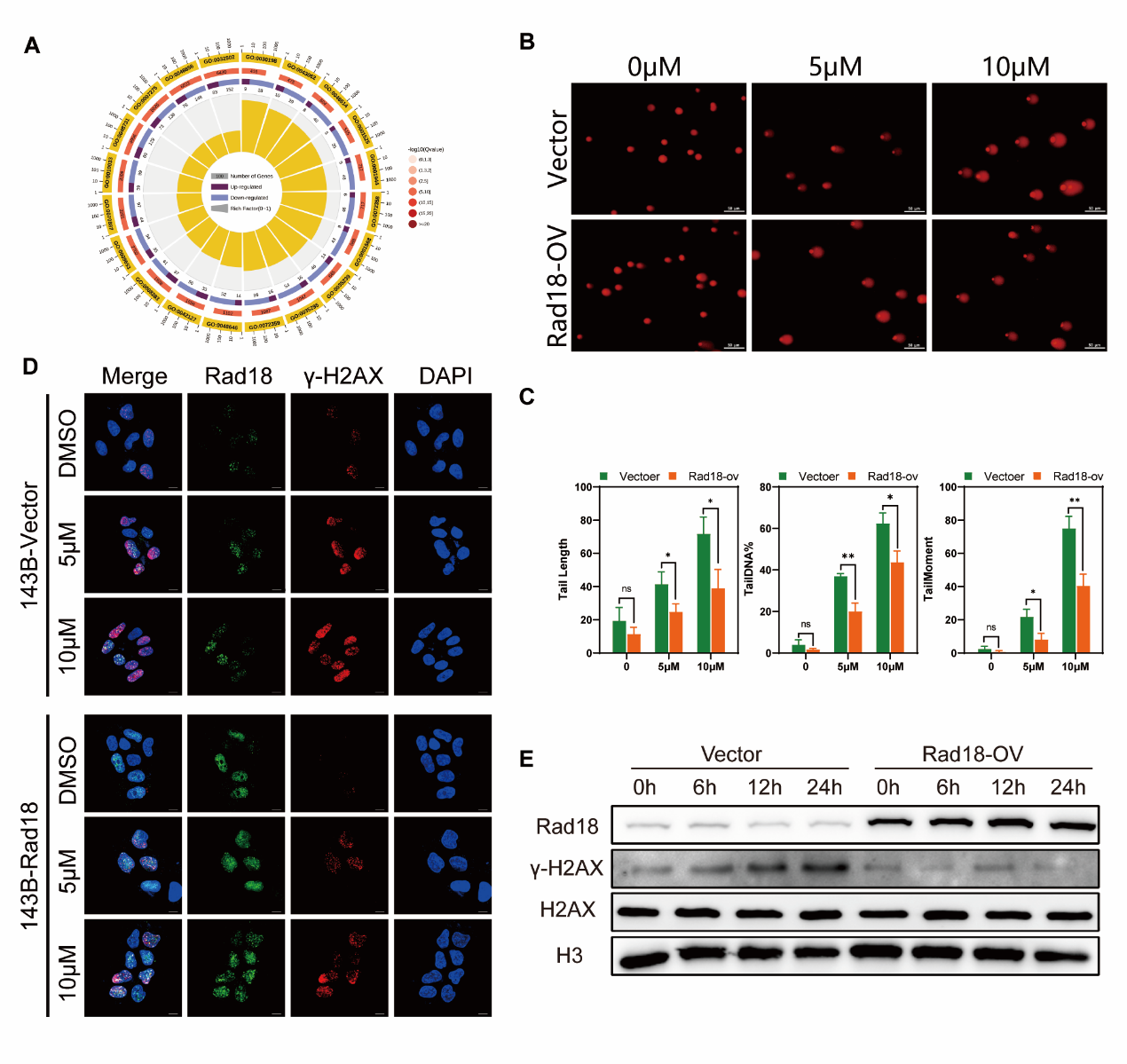


**Supplementary Fig. 4 (Related to Fig. 4)**

**A.** Results of RNA sequencing of 143B and Rad18-knockout 143B cells. **B.** Doxorubicin-induced DNA damage was measured by comet assay in Rad18 overexpressed cells. Scale bar: 50µm. **C.** DNA comet tail percent, tail moment and tail length were measured in Rad18 overexpressed cells. **D.** Immunofluorescence staining of in 143B and Rad18 overexpressed 143B cells with γ-H2AX and Rad18 antibodies. Scale bar =10 μm. **E.** Western blot analysis of γ-H2AX and Rad18 in 143B and Rad18 overexpressed 143B cells with 5μM doxorubicin treatment for 0h, 6h, 12h and 24h.


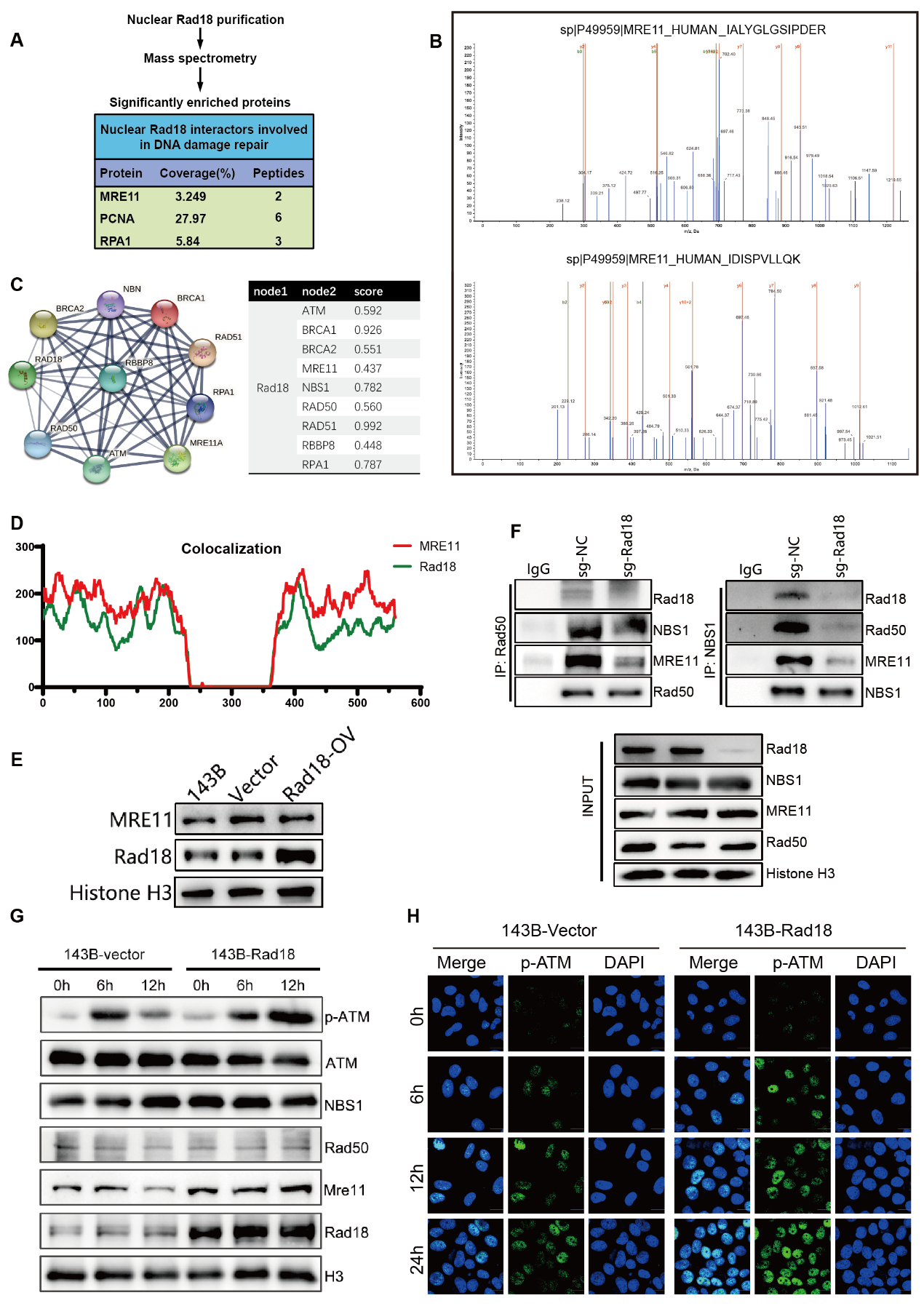


**Supplementary Fig. 5 (Related to Fig. 5)**

**A.** Two peptides were identified originating from MRE11 by secondary spectra. **B.** STRING database predicted the relationship between Rad18 and MRE11 and homologous recombination repair pathways. **C. and D.** Fluorescence intensity regression analysis showed the colocalization of Rad18 and MRE11. **E.** The expression level of MRE11 was measured by western blotting after overexpression of Rad18. **F.** Co-IP showed that after Rad18 was knocked out, the integration of MRN complex was reduced, rather than the expression of the individual component. **G.** The change of ATM phosphorylation was determined after Rad18 was overexpressed by western blotting. **H.** Immunofluorescence was utilized to monitor the change of ATM phosphorylation after Rad18 overexpression. Scale bar: 10 µm.


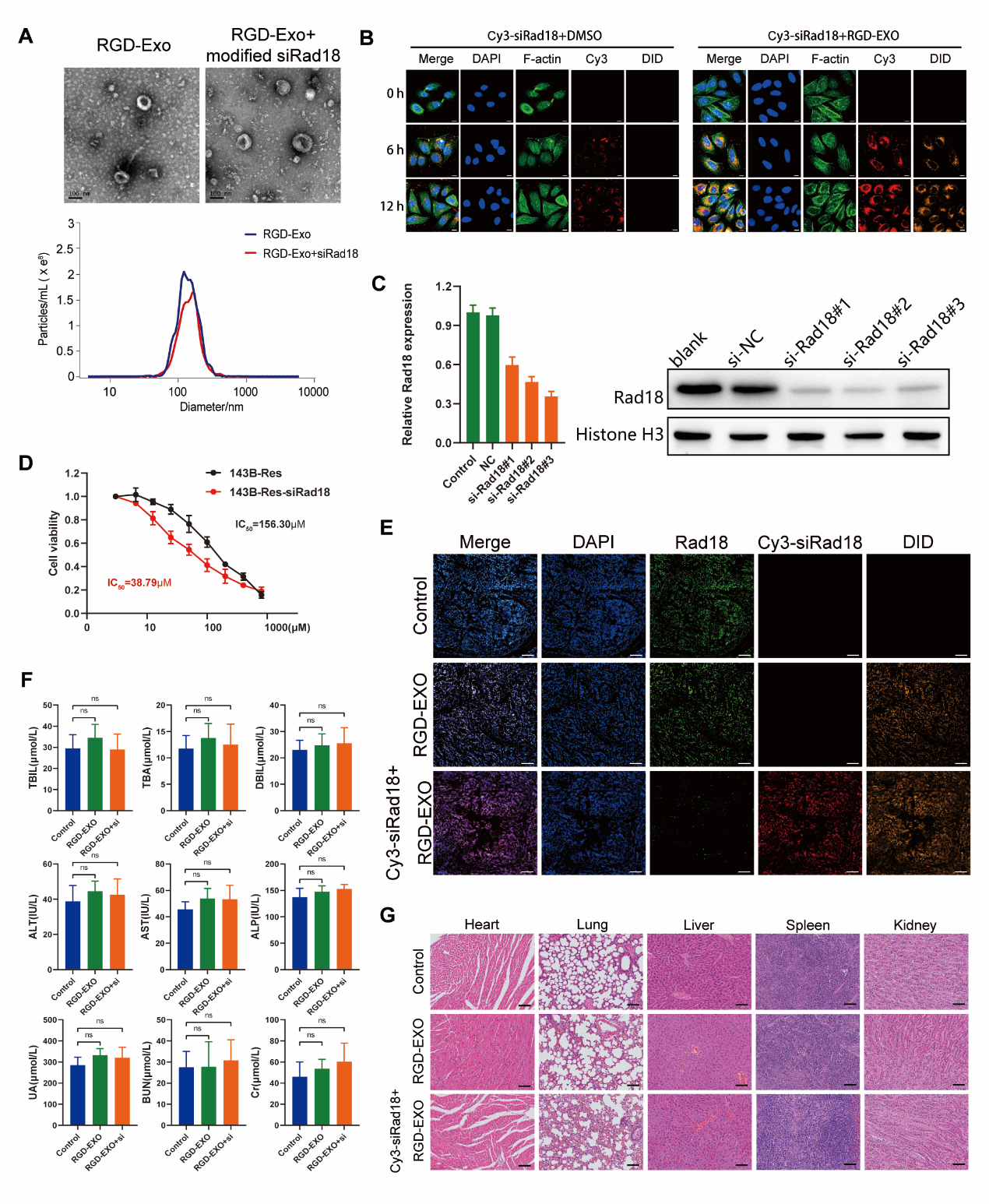


**Supplementary Fig. 6 (Related to Fig. 6)**

**A.** Electron microscope identification found no obvious morphological changes and little difference in particle size between the RGD-Exo loaded with siRad18 and the empty RGD-Exo. Scale bar: 100 nm. **B.** Confocal images of 143B cells transfected with cholesterol-cy3-siRad18-loaded RGD-EXOs or cholesterol-modified-siRad18, showing the relative localization of the cy3-siRad18 (red), F-actin(green) and RGD-EXOs stained with DID (orange) at incubation times of 0h, 6h and 12h respectively. Cell nuclei were stained with DAPI (blue). Scale bar =10 μm. **C.** The cholesterol modified siRad18 delivered by engineered exosomes reduced mRNA and protein expression of Rad18. **D.** The change sensitivity to doxorubicin in cells after Rad18-knockdown. **E.** The distribution of RGD exosomes with or without modified-siRad18 was detected in vivo. **F.** liver and kidney function analysis showed that control RGD-EXO and RGD-EXO -siRad18 had no significant effect on liver and kidney function of nude mice. **G.** HE sections of all organs showed no tissue and organ damage.

**Key resources table**

| REAGENT or RESOURCE | SOURCE | IDENTIFIER |
| --- | --- | --- |
| Antibodies |  |  |
| Anti-Rad18 | Abcam | Cat#ab188235 |
| Anti-Histone H3 | Cell Signaling Technology | Cat# #4499 |
| Anti-γ-H2AX | Abcam | Cat#ab26350 |
| Anti-H2AX | Abcam | Cat#ab229914 |
| Anti-p-ATM | Abcam | Cat#ab81292 |
| Anti- ATM | Abcam | Cat#ab32420 |
| Anti-MRE11 | Abcam | Cat#ab208020* |
| Anti-NBS1 | Abcam | Cat#ab32074 |
| Anti-Rad50 | Abcam | Cat#ab228886 |
| Anti-Caspase/3 | Abcam | Cat#51064-2-AP |
| Anti-FLAG tag | Proteintech | Cat#20543-1-AP |
| Anti-HA tag | Proteintech | Cat#ab8880 |
| CoraLite®488-Phalloidin (green) | Proteintech | PF00001 |
| CoraLite488-conjugated Goat Anti-Mouse IgG(H+L) | Proteintech | Cat#SA00013-1 |
| CoraLite488-conjugated Goat Anti-Rabbit IgG(H+L) | Proteintech | Cat#SA00013-2 |
| Cy3–conjugated Affinipure Goat Anti-Mouse IgG(H+L) | Proteintech | Cat#SA00009-1 |
| Cy3–conjugated Affinipure Goat Anti-Mouse IgG(H+L) | Proteintech | Cat#SA00009-2 |
| CoraLite488-conjugated Goat Anti-Mouse IgG(H+L) | Proteintech | Cat#SA00013-3 |
| CoraLite488-conjugated Goat Anti-Rabbit IgG(H+L) | Proteintech | Cat#SA00013-4 |
| HRP Goat Anti-Rabbit IgG | Servicebio | GB23303 |
| HRP Goat Anti-Mouse IgG | Servicebio | GB23301 |
| HRP Goat Anti-Rabbit IgG | Abcam | ab6721 |
| HRP Goat Anti-Mouse IgG | Abcam | ab205719 |
| Chemicals, Peptides, and Recombinant Proteins |  |  |
| DMSO | Sigma-Aldrich | Cat# D8418 |
| Trypsin | Life Technologies | Cat#15090046 |
| Matrigel | BD Biosciences | Cat#356234 |
| Luciferin | Biocat | Cat#7903-100-BV |
| Poly-L-lysine | Sigma-Aldrich | Cat#P5899 |
| Lipofectamine ^®^ 3000 | Invitrogen | Cat#L3000001 |
| PrimeScript^TM^ RT Master Mix | Takara | Cat#RR036A |
| PrimeScript^TM^ RT Master Mix | Takara | Cat# RR037A |
| TrypLE^TM^ Express Enzyme | ThermoFisher Scientific | Cat#12605010 |
| Fetal bovine serum | ThermoFisher Scientific | Cat# 10438018 |
| Fast SYBR^®^ Green Master Mix | Applied Biosystems | Cat#4385612 |
| Penicillin-Streptomycin | ThermoFisher Scientific | Cat#15140122 |
| Polybrene | Sigma-Aldrich | Cat#TR-1003 |
| Critical Commercial Assays |  |  |
| PE Annexin V Apoptosis Detection Kit I | BD Biosciences | Cat#559763 |
| Pierce™ Rapid Gold BCA Protein Assay Kit | ThermoFisher Scientific | Cat#A53225 |
| Experimental Models: Cell Lines |  |  |
| 143B | ATCC | CRL-8303 |
| Saos-2 | ATCC | HTB-85 |
| MG-63 | ATCC | CRL-1427 |
| U-2 OS | ATCC | HTB-96 |
| HOS | ATCC | CRL-1543 |
| hFOB 1.19 | Procell | CL-0353 |
| OS#1 | This study | N/A |
| Experimental Models: |  |  |
| Balb/c nude mice | Laboratory Animal Center, Fourth Military Medical University | N/A |
| Software and Algorithms |  |  |
| ImageJ | https://imagej.nih.gov/ij/ | N/A |
| GraphPad Prism 7 | GraphPad Software | N/A |
| R package | http://cran.us.r-project.org | N/A |
| FlowJo 10.0 | TreeStar | N/A |
| Deposited Data |  |  |
| Gene Expression Profiling Interactive Analysis | <http://gepia.cancer-pku.cn/)> | GEPIA |
| GSE39058, GSE87437 | https://www.ncbi.nlm.nih.gov/pmc/ | GEO |
| STRING | https://string-db.org/ | STRING |
|  |  |  |

[**Ncleotide**](javascript:;) [**sequence**](javascript:;)

| Gene | siRNA | sequence |
| --- | --- | --- |
| ID: 56852  human-RAD18 | siRNA#1 | sense（5'-3'）CAUUGCAAUGAUAAUACCUTT |
|  |  | antisense（5'-3'）AGGUAUUAUCAUUGCAAUGTT |
|  | siRNA#2 | sense（5'-3'）GCUGUAUUGAUAUUCAAGATT |
|  |  | antisense（5'-3'）UCUUGAAUAUCAAUACAGCTT |
|  | siRNA#3 | sense（5'-3'）CCAGAAAGUCACAUUAAUATT |
|  |  | antisense（5'-3'）UAUUAAUGUGACUUUCUGGTT |
| ID: 51704  human-GPRC5B | siRNA#1 | sense（5'-3'）CCAUGUACCUCUUCGGCAATT |
|  |  | antisense（5'-3'）UUGCCGAAGAGGUACAUGGTT |
|  | siRNA#2 | sense（5'-3'）CGUUUAGAAGCAACGUGUATT |
|  |  | antisense（5'-3'）UACACGUUGCUUCUAAACGTT |
|  | siRNA#3 | sense（5'-3'）GCCCUCAUCUACGACAUGGUA |
|  |  | antisense（5'-3'）UACCAUGUCGUAGAUGAGGGC |
| ID: 547  human-KIF1A | siRNA#1 | sense（5'-3'）UAAAGCAUUAAAUGAAACCAU |
|  |  | antisense（5'-3'）GGUUUCAUUUAAUGCUUUAUA |
|  | siRNA#2 | sense（5'-3'）AUUUAUUUCUUAAUUGGACAG |
|  |  | antisense（5'-3'）GUCCAAUUAAGAAAUAAAUGU |
|  | siRNA#3 | sense（5'-3'）UUUUGUGUUGAGUAAGAGCCA |
|  |  | antisense（5'-3'）GCUCUUACUCAACACAAAAAA |
| ID: 121643  human-FOXN4 | siRNA#1 | sense（5'-3'） AUUUUUCACAUUUGACUAGCU |
|  |  | antisense（5'-3'） CUAGUCAAAUGUGAAAAAUGC |
|  | siRNA#2 | sense（5'-3'） UGUGAAUAACAGUUUCAACAU |
|  |  | antisense（5'-3'） GUUGAAACUGUUAUUCACAAA |
|  | siRNA#3 | sense（5'-3'） UUGAUGUUGCCCAAACACGGA |
|  |  | antisense（5'-3'）UUGAUGUUGCCCAAACACGGA |

**Primer sequence**

| human-RAD18 | FORWARD——ATTGGAACCTGACAGAGAAGAG |
| --- | --- |
|  | REVERSE——CCAGGCTTCCTCTTCTTCTAAA |
| human-KIF1A | FORWARD——AAGAGGGATGTAAAGTTCACGT |
|  | REVERSE——CTTGAAAAACACTCGAGCAGAA |
| human-FOXN4 | FORWARD——GATATCACCAGCGAGTTGAGTA |
|  | REVERSE——CTCTCAATTTTTCCATAGCCCG |
| human-GPRC5B | FORWARD——GTGACATTTCTCACGATGCTAC |
|  | REVERSE——TTCTGCAGTGTTCTATCAGAGG |
